# Prognostic Impact of Implementation of Heart Team Approach on Cardiovascular Outcomes in Patients With Complex Coronary Artery Disease

**DOI:** 10.1002/clc.70141

**Published:** 2025-05-02

**Authors:** Po‐Hsueh Su, Ya‐Lin Huang, Po‐Wei Chen, Hsien‐Yuan Chang, Jun‐Neng Roan, Ting‐Hsing Chao

**Affiliations:** ^1^ Department of Internal Medicine, Division of Cardiology National Cheng Kung University Hospital, College of Medicine, National Cheng Kung University Tainan Taiwan; ^2^ Department of Internal Medicine, Division of Cardiology Madou Sin‐Lau Hospital Tainan Taiwan; ^3^ Department of Surgery, Division of Cardiovascular Surgery National Cheng Kung University Hospital, College of Medicine, National Cheng Kung University Tainan Taiwan; ^4^ School of Medicine, College of Medicine National Cheng Kung University Tainan Taiwan; ^5^ Department of Internal Medicine, Division of Cardiology Chung‐Shan Medical University Hospital and School of Medicine, Chung Shan Medical University Taichung Taiwan

**Keywords:** coronary artery bypass grafting, coronary artery disease, percutaneous coronary intervention, quality improvement

## Abstract

**Background:**

In patients with stable coronary artery disease (CAD), treatment options include medical therapy, percutaneous coronary intervention (PCI), and coronary artery bypass grafting (CABG). The decision between PCI and CABG depends on disease severity and revascularization risk. Guidelines promote a heart team approach (HTA) with shared decision‐making, yet PCI remains prevalent.

**Methods and Results:**

We conducted a retrospective analysis of 753 patients with complex CAD (left main or multivessel disease and SYNTAX score ≥ 33) between January 2019 and April 2022. We evaluated a quality improvement program featuring a clinical decision flow map, a support system for risk score calculations, and a standard operating procedure for HTA. We compared HTA activation, revascularization strategy choices, and long‐term cardiovascular outcomes (composite endpoint: death, myocardial infarction, or unplanned revascularization) between patients treated with HTA (HTA group, *n* = 448) and without HTA (non‐HTA group, *n* = 304). The program significantly increased HTA activation (from 26.4% to 61.7%) and CABG selection (from 11.1% to 20.4%). The HTA group had better CABG recommendations and choices (75.5% vs. 37.2%, and 26.8% vs. 7.2%, respectively), with a lower incidence of the primary composite endpoint (18.1% vs. 42.4%).

**Conclusion:**

A quality improvement program enhances HTA activation and revascularization strategies, leading to improved cardiovascular outcomes in complex CAD patients.

## Graphical abstract

Implementation of the quality improvement program significantly enhanced HTA activation, led to a significant increase in the selection of CABG as a treatment option. In addition, HTA activation reduced primary composite endpoint after adjustment for confounders.

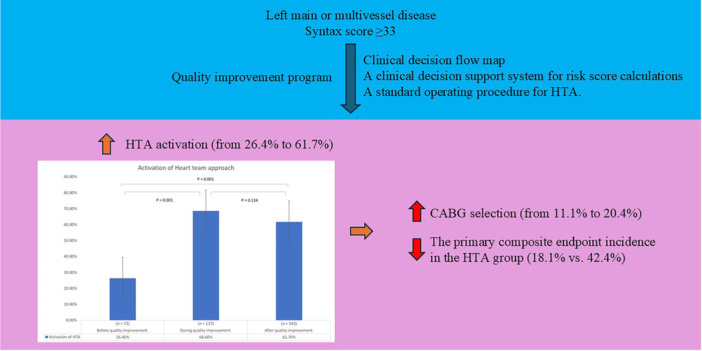

## 1 Introduction

Treatment strategies for patients with stable coronary artery disease (CAD), also referred to as chronic coronary syndrome, include medical therapy, percutaneous coronary intervention (PCI), and coronary artery bypass grafting (CABG) [1, 2, 3]. All of these approaches can alleviate anginal symptoms; however, only a small subset of patients derive cardiovascular benefits, such as a decreased risk of death or myocardial infarction, from coronary revascularization (PCI or CABG) compared with medical therapy alone [1, 2, 3]. Several factors—such as disease severity, comorbidities, and the feasibility of or risks associated with revascularization—should be considered when selecting between PCI and CABG [4]. The SYNTAX (SYNergy between PCI with TAXUS and Cardiac Surgery) score is a validated tool widely recommended for assessing CAD severity and helping guide revascularization strategy in patients with multivessel or left main disease [1, 2, 5, 6]. Similarly, the EuroSCORE II is commonly used to evaluate cardiac surgery risks [7]. Of the therapies available, PCI is more frequently chosen in clinical practice because of its minimally invasive nature, its shorter procedure time, and the advancements made in diagnostic and intervention devices, even for patients with complex CAD [8]. Ad hoc PCI is also commonly performed. To address potential referral biases and improve adherence to guidelines, international recommendations strongly advocate the heart team approach (HTA) with shared decision‐making for cases of complex CAD to ensure that treatment decisions align with the patient's preferences, needs, and values [1, 2, 9].

The heart team operates as a multidisciplinary dialogue between specialists with the aim of delivering optimal, patient‐centered care [9, 10]. Interdisciplinary heart team discussions can improve appropriate PCI use [10, 11], provide superior assessments for planning CABG success [12], and offer reproducible, consistent recommendations for patients with complex CAD [10, 13, 14]. However, to the best of our knowledge, no studies have directly compared the cardiovascular outcomes of patients with complex CAD who were treated using the HTA versus those who were not. Studies have only demonstrated the superiority of CABG over PCI or medical therapy in terms of intermediate or long‐term outcomes for patients treated using the HTA [15, 16, 17, 18]. Recently, Witberg et al found that patients undergoing CABG for whom the heart team recommendation diverged from the guideline recommendation had poorer cardiovascular outcomes than those for whom a concordant decision was made [10]. In the present study, we retrospectively evaluated a prospective cohort enrolled in the case management program of the disease‐specific care certification for CAD at our hospital. We investigated the effect of a comprehensive quality improvement activity on HTA activation and revascularization choice in patients with left main disease or a SYNTAX score of ≥ 33. In addition, we evaluated long‐term cardiovascular outcomes in these patients, comparing those who were treated through the HTA with those who were not. We expected that the quality improvement activity would increase the likelihood of HTA activation, lead to changes in revascularization patterns, and result in better cardiovascular outcomes for patients with complex CAD.

## 2 Methods

### 2.1 Patient Population

This retrospective study included a prospective cohort of 753 patients who were consecutively enrolled in the case management program for disease‐specific care certification for CAD between January 2019 and April 2022 and who received a diagnosis of complex CAD through invasive coronary angiography (ICA). CAD was defined as substantial luminal narrowing (≥ 50% diameter stenosis compared with the adjacent reference vessel) in at least one coronary artery (left main trunk, left anterior descending artery, left circumflex artery, or right coronary artery) or a major branch (diagonal branch, obtuse marginal branch, acute marginal branch, posterior descending artery, or posterior lateral branch), as observed through ICA. Complex CAD was defined as substantial luminal narrowing in the left main trunk (left main disease) or in 2 or more coronary arteries or major branches (multivessel disease).

The case management program for disease‐specific care certification for CAD has been operational in our hospital since 2014. We have been prospectively enrolling hospitalized patients with ICA‐confirmed CAD into this program for comprehensive interdisciplinary care. Data are collected to report quality indicators and are routinely reviewed during monthly team meetings.

Given the retrospective nature of the present study, informed consent was not required to prevent selection bias. This study was approved by the Institutional Review Board of National Cheng Kung University (identifier: A‐ER‐111‐263).

### 2.2 Implementation of a Comprehensive Quality Improvement Program

Between April and September 2019, we implemented a comprehensive quality improvement initiative aimed at enhancing use of the HTA in patients with complex CAD due to previous suboptimal performance.

The initiative involved reviewing contemporary evidence and guidelines, establishing a clinical decision flow map, introducing a clinical decision support system to mandate SYNTAX score and EuroSCORE calculations, and developing a standard operating procedure for heart team activation.

#### 2.2.1 HTA

The HTA was structured and operated in line with previous recommendations [9]. In brief, a patient's SYNTAX score was calculated immediately after ICA was performed and communicated to the operators by trained technicians. For patients meeting the criteria for complex CAD, the operators strongly advised both the patient and their family to avoid ad hoc PCI and proceed with the HTA. In‐charge cardiac surgeons were notified, and heart team discussions, typically scheduled between 16:30 and 17:00, were held in which interventionists and surgeons met to discuss the case. The EuroSCORE was also calculated during this process. The outcomes of the heart team discussion were documented in the meeting minutes signed by both cardiac surgeons and interventionists and provided to the patient and their family, along with decision aids to facilitate shared decision‐making for selecting the coronary revascularization strategy. In certain cases, if the patient wished to make an immediate decision, the in‐charge cardiac surgeon was called to the catheterization laboratory for an on‐table discussion.

Patients who participated in the HTA were categorized into the HTA group, whereas those who declined were assigned to the non‐HTA group.

### 2.3 Collection of Background Characteristics and Clinical Outcomes

Background characteristics were collected through a review of medical records. The primary composite endpoint was death, myocardial infarction, or unplanned coronary revascularization during long‐term follow‐up. The prespecified secondary endpoints were the individual components of the primary composite endpoint. Clinical outcomes were obtained through medical record reviews. The detailed criteria for diagnosing myocardial infarction and unplanned coronary revascularization have been described previously [19].

### 2.4 Statistical Analysis

Continuous variables are expressed as the mean ± standard deviation, whereas skewed data are reported as the median (interquartile range). The two group's categorical variables were compared using the chi‐square or Fisher's exact test at different time points, whereas continuous variables were compared using the Mann–Whitney *U* test or unpaired Student's *t* test as appropriate. If more than one event occurred during the follow‐up period, only the first event was considered in the analysis. Kaplan–Meier analysis was performed to assess patient survival and event‐free status, and differences between the groups were determined using the log‐rank test (Cox–Mantel). A P value of < 0.05 was considered statistically significant. All statistical analyses were performed using SPSS 23 for Windows (IBM, Armonk, NY, USA).

## 3 Results

**Figure 1:**
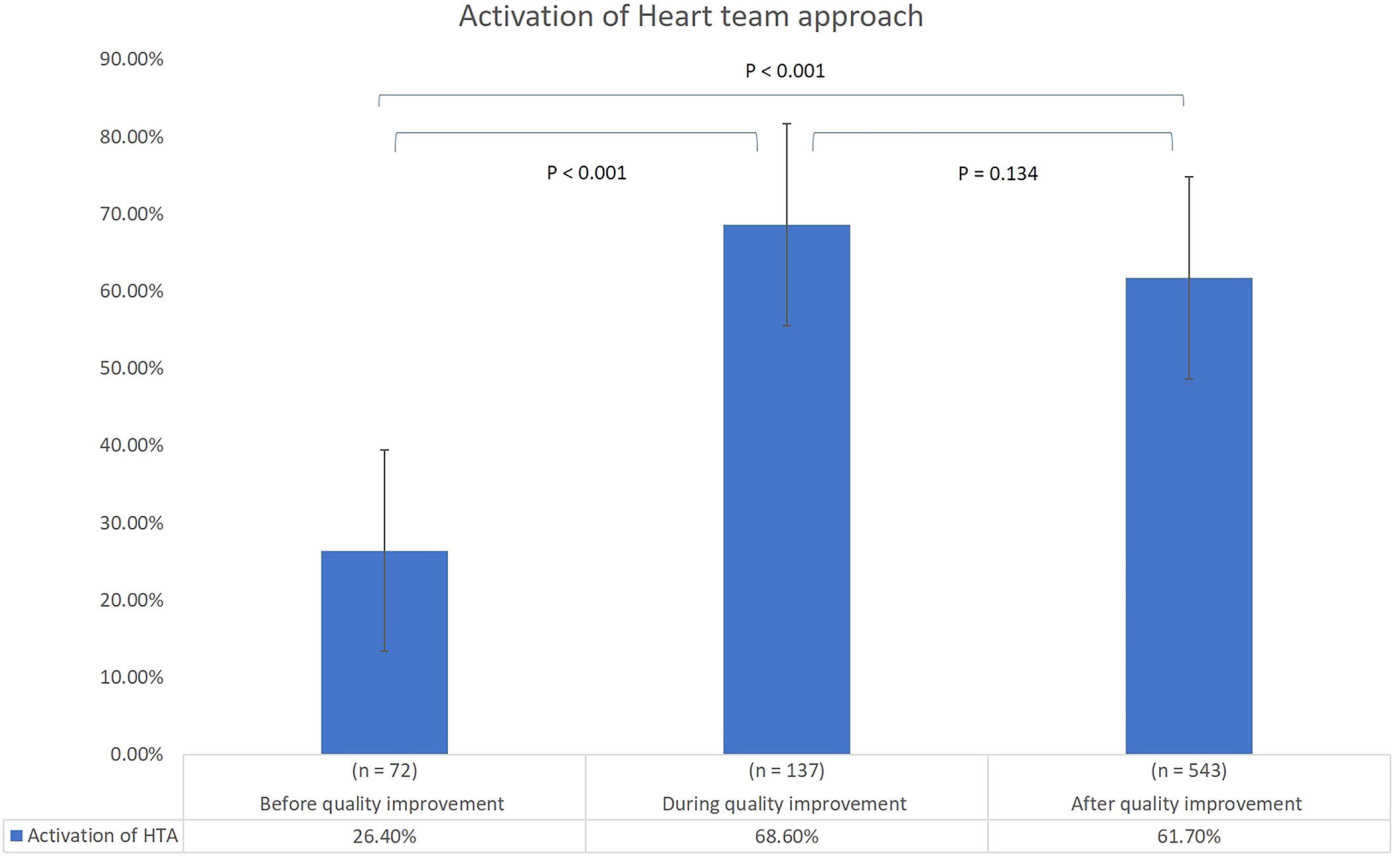
Effects of the implementation of a quality improvement program on HTA activation. HTA, heart team approach.

Implementation of the quality improvement program significantly enhanced HTA activation (from 26.4% before the program to 68.6% during and 61.7% after, *p* < 0.001; Figure 1, Table 1). This program also led to a significant increase in the selection of CABG as a treatment option (from 11.1% before the program to 16.8% during and 20.4% after, *p* = 0.006; Table 2).

**Table 1:** HTA activation during different time periods with respect to implementation of the quality improvement program.

	Before quality improvement (*n* = 72)	During quality improvement (*n* = 137)	After quality improvement (*n* = 543)	*P* value
Activation of HTA	26.4%	68.6%	61.7%	< 0.001

Abbreviation: HTA, heart team approach.

**Table 2:** Choice of revascularization strategy during different time periods with respect to implementation of the quality improvement program.

	Before quality improvement (*n* = 72)	During quality improvement (*n* = 137)	After quality improvement (*n* = 543)	P value
CABG	11.1%	16.8%	20.4%	0.006
PCI	76.4%	75.2%	68.9%	
Hybrid (PCI followed by CABG)	2.8%	1.5%	0	
Medical treatment	9.7%	6.6%	10.7%	

Abbreviations: CABG, coronary artery bypass grafting; PCI, percutaneous coronary intervention.

Table 3 presents the background characteristics and clinical and angiographic parameters of the HTA and non‐HTA groups. Patients in the HTA group (*n* = 448) were younger, had a lower EuroSCORE II score and a lower rate of previous PCI or CABG, and were more likely to have three‐vessel disease than those in the non‐HTA group (*n* = 304). In addition, the HTA group had a higher percentage of patients with three‐vessel disease and smoking history. The use of drug‐eluting stents was less frequent in the HTA group.

**Table 3:** Baseline characteristics and clinical and angiographic parameters in the HTA and non‐HTA groups.

	HTA (*n* = 448)	Non‐HTA (*n* = 304)	*P* value
Age, yr	66.8 ± 10.7	68.9 ± 11.0	0.009
Male gender	78.8%	77.6%	0.704
Left main disease	49.1%	44.7%	0.239
TVD	99.6%	97.7%	0.035
SYNTAX score	41.7 ± 7.7	40.8 ± 7.7	0.122
EuroSCORE II	3.6 ± 6.1	5.4 ± 9.7	0.010
ACS	40.4%	40.5%	0.987
Cigarette smoking	21.2%	13.5%	0.007
Diabetes mellitus	67.2%	63.5%	0.294
Hypertension	73.0%	72.0%	0.774
Hyperlipidemia	66.1%	67.4%	0.697
History of CVA	11.6%	13.5%	0.442
History of PAD	7.4%	9.5%	0.288
History of old MI	11.6%	14.5%	0.248
Previous PCI	24.3%	36.8%	< 0.001
Previous CABG	2.9%	15.5%	< 0.001
LV systolic dysfunction	20.3%	15.8%	0.117
CKD	37.5%	35.2%	0.520
ESRD	21.7%	23.0%	0.656

Abbreviations: ACS, acute coronary syndrome; CABG, coronary artery bypass grafting; CKD, chronic kidney disease; CVA, cerebrovascular accident; ESRD, end‐stage renal disease; HTA, heart team approach; LV, left ventricular; MI, myocardial infarction; PAD, peripheral artery disease; PCI, percutaneous coronary intervention; TVD, three‐vessel disease.

Physicians initially recommended CABG more frequently, and the final decision to proceed with CABG was more common in the HTA group than in the non‐HTA group (75.5% vs. 37.2% and 26.8% vs. 7.2%, respectively; both *p* < 0.001). However, PCI remained the most chosen strategy overall (Table 4).

**Table 4:** Treatment strategy in the HTA and non‐HTA groups.

	HTA (*n* = 448)	Non‐HTA (*n* = 304)	*P* value
Initial recommendation			
CABG	75.5%	37.2%	< 0.001
PCI	21.0%	57.2%	
PCI or CABG	1.6%	0.7%	
Medical	2.2%	4.9%	
Final choice			
CABG	26.8%	7.2%	< 0.001
PCI	62.7%	82.6%	
Hybrid (PCI followed by CABG)	0.2%	1.0%	
Medical	10.3%	9.2%	
Deployment of DES	51.3%	67.4%	< 0.001

Abbreviations: CABG, coronary artery bypass grafting; DES, drug‐eluting stent; HTA, heart team approach; PCI, percutaneous coronary intervention.

During a mean follow‐up period of 487.14 ± 377.69 days, the primary composite endpoint occurred in 213 patients. The HTA group had a significantly lower risk of the primary composite endpoint and lower incidence of total death, myocardial infarction, and unplanned coronary revascularization than did the non‐HTA group (18.1% vs. 42.4%, *p* < 0.001; 10.9% vs. 21.7%, *p* < 0.001; 3.8% vs. 9.2%, *p* = 0.002; and 8.7% vs. 22.7%, *p* < 0.001, respectively; Table 5). However, the risk of stroke was similar in the two groups.

**Table 5:** Clinical outcomes of the HTA and non‐HTA groups.

	HTA (*n* = 448)	Non‐HTA (*n* = 304)	*P* value
Primary composite endpoint	18.1%	42.4%	< 0.001
Secondary endpoints			
Total death	10.9%	21.7%	< 0.001
Myocardial infarction	3.8%	9.2%	0.002
Unplanned coronary revascularization	8.7%	22.7%	< 0.001

Abbreviation: HTA, heart team approach.

To identify independent predictors of the primary composite endpoint, univariate and multivariate analyses were performed (Table 6). HTA activation remained an independent predictor of the primary composite endpoint after adjustment for confounders (hazard ratio [HR] 0.514, 95% confidence interval [CI] 0.375–0.704). The other independent factors were age (HR 1.020, 95% CI: 1.005–1.036), SYNTAX score (HR: 1.031, 95% CI: 1.013–1.050), EuroSCORE II score (HR: 1.024. 95% CI: 1.011–1.037), diabetes mellitus (HR: 1.523, 95% CI: 1.050–2.210), hyperlipidemia (HR: 0.609, 95% CI: 0.448–0.828), end‐stage renal disease (HR: 2.647, 95% CI: 1.871–3.764), and the final choice of CABG (HR: 0.343, 95% CI: 0.186–0.632). (Graphic abstract)

**Table 6:** Predictors of the primary composite endpoint, determined through univariate and multivariate analyses.

	Uni‐variate analysis		Multi‐variate analysis	
	OR (95% CI)	*P* value	OR (95% CI)	*P* value
HTA	0.381 (0.289–0.504)	< 0.001	0.514 (0.375–0.704)	< 0.001
Age	1.027 (1.014–1.041)	< 0.001	1.020 (1.005–1.036)	0.011
Male gender	0.741 (0.545–1.006)	0.054	—	NS
Left main disease	1.299 (0.990–1.704)	0.059	—	NS
TVD	1.561 (0.388–6.284)	0.531		
SYNTAX score	1.015 (0.998–1.032)	0.091	1.031 (1.013–1.050)	0.001
EuroSCORE II	1.028 (1.019–1.038)	< 0.001	1.024 (1.011–1.037)	< 0.001
ACS	1.189 (0.906–1.560)	0.213		
Cigarette smoking	0.470 (0.302–0.732)	0.001	—	NS
Diabetes mellitus	1.411 (1.039–1.917)	0.027	1.523 (1.050–2.210)	0.027
Hypertension	0.856 (0.634–1.157)	0.313		
Hyperlipidemia	0.577 (0.436–0.762)	< 0.001	0.609 (0.448–0.828)	0.002
History of CVA	1.620 (1.122–2.339)	0.010	—	NS
History of PAD	2.807 (1.951–4.040)	< 0.001	—	NS
History of old MI	1.489 (1.039–2.132)	0.030	—	NS
Previous PCI	1.924 (1.463–2.530)	< 0.001	—	NS
Previous CABG	1.404 (0.918–2.149)	0.118	—	
LV systolic dysfunction	1.399 (1.009–1.941)	0.044	—	NS
CKD	1.670 (1.273–2.191)	< 0.001	—	NS
ESRD	2.754 (2.075–0.656)	< 0.001	2.647 (1.871–3.764)	< 0.001
Final choice of CABG	0.245 (0.140–0.430)	< 0.001	0.343 (0.186–0.632)	0.001
DES deployment	1.112 (0.841–1.470)	0.457		

Abbreviations: ACS, acute coronary syndrome; CABG, coronary artery bypass grafting; CI, confidence interval; CKD, chronic kidney disease; CVA, cerebrovascular accident; DES, drug‐eluting stent; ESRD, end‐stage renal disease; HTA, heart team approach; LV, left ventricular; MI, myocardial infarction; OR, odds ratio; PAD, peripheral artery disease; PCI, percutaneous coronary intervention; TVD, three‐vessel disease.

## 4 Discussion

The current study demonstrated that a comprehensive quality improvement program significantly increased HTA activation and affected patients’ revascularization strategy choices. HTA was found to be independently associated with a lower risk of death, myocardial infarction, or unplanned coronary revascularization in patients with complex CAD. To the best of our knowledge, no other studies have reported similar findings. These results provide strong support for recommendations from both international and local societies advocating for the integration of the HTA into the management of complex CAD. This aligns with the core principle that collaborative, interdisciplinary discussions lead to more tailored, patient‐centric care strategies that address the multifaceted nature of CAD.

International guidelines strongly recommend the HTA with shared decision‐making for selecting a revascularization strategy in cases of complex CAD [1, 2, 9]. However, to the best of our knowledge, this recommendation is solely based on expert opinion instead of solid evidence. No studies, whether prospective or observational, have directly compared cardiovascular outcomes between patients with complex CAD who receive the HTA versus those who do not. Other studies have only indicated that CABG is superior to PCI or medical therapy in terms of intermediate or long‐term outcomes in patients who are treated using the HTA [15, 16, 17, 18]. Our study provides evidence supporting the guideline recommendation. Consistent with previous findings, the results of the current study demonstrated that the HTA increases the likelihood of CABG selection [10, 11].

Our study revealed that both the HTA and the final choice of CABG were independent predictors of the primary composite endpoint, suggesting that the benefits of the HTA were not solely attributable to the increased use of CABG. The potential mechanisms underlying improved cardiovascular outcomes with the HTA in patients with complex CAD are as follows: 1) HTA facilitates superior assessment and planning for CABG, which is considered superior or, at the very least, noninferior to PCI or medical treatment in most patients with complex CAD in the current era [1, 2, 3]. 2) Interdisciplinary discussions yield optimal management recommendations because the risks and benefits of each strategy are fully considered on a patient‐centered basis. Such discussion ensures that decisions regarding revascularization strategies are made with a comprehensive understanding of each patient's unique clinical scenario, thereby improving the graft success rate and overall patient outcomes. 3) The advantages of the HTA extend beyond the optimal selection of a revascularization strategy. The multidisciplinary approach also involves patient education, shared decision‐making, and coordinated care—all key functions of the heart team that contribute to improved patient outcomes.

Although the HTA is strongly recommended for patients with complex CAD, the infrastructure and execution of heart teams in different institutions vary considerably [20], and inappropriate use of PCI remains common [21]. A significant gap persists between evidence‐based clinical guidelines and real‐world practice [22]. The current study demonstrated that implementing a comprehensive quality improvement program could enhance HTA activation and should be considered in institutions with a low HTA execution rate. The positive impact of this program, as demonstrated in this study, highlights the value of structured, team‐based care models in improving the standard of care for patients with complex CAD. Despite improvements in technology, PCI was unable to reduce the gap between optimal medical treatment and CABG in all randomized clinical trials performed in the last 25 years including those at low CAD risk as was seen in the recently published FAME 3 study [23].

To address the gap between guidelines and practice, understanding the behavior of interventionists, patients, and families when choosing a revascularization strategy is crucial, despite the lack of previous reports on this topic. The background characteristics of the HTA and non‐HTA groups of the current study offer valuable insights. The data indicate that physicians may forgo the HTA (or the patient or family may opt for ad hoc PCI) in cases where the patient is at high surgical risk (e.g., those with a high EUROSCORE II score, older age, or history of PCI or CABG). Encouraging physicians, patients, and families to proceed with the HTA instead of ad hoc PCI after ICA could help enhance HTA activation, especially for patients for whom the risk posed by surgery is high.

Although patients’ final choice of revascularization often differed from the heart team's initial recommendation, we noted an improvement in cardiovascular outcomes. This improvement is likely due to the increased selection of CABG and the comprehensive care provided by the team. The discrepancy between the initial recommendation and final treatment decision highlights the complexity of clinical decision‐making and the importance of engaging patients and their families in the process.

The present study has some limitations that should be addressed. The data were derived from a single center and involved a relatively small sample, which may have limited the generalizability of the findings, although our cohort was larger than those in other studies [15, 16, 18]. Potential biases may not have been excluded owing to the retrospective nature of the current study. Although we performed multivariate analysis, some potential confounders, including unknown variables, may not have been fully accounted for.

## 5 Conclusions

The present study provides clinical evidence supporting the effectiveness of a comprehensive quality improvement program, demonstrating that the HTA can result in better cardiovascular outcomes in patients with complex CAD. The HTA should be implemented in clinical practice. Our results highlight the need for further investigation into the effects of the HTA in a prospective randomized controlled trial.

## Conflicts of Interest

The authors declare no conflicts of interest.

## Data Availability

The data underlying this article will be shared on reasonable request to the corresponding author.
